# Introducing competency-based postgraduate medical training: gains and losses

**DOI:** 10.5116/ijme.4e78.427f

**Published:** 2011-10-03

**Authors:** Niels Kristian Kjaer, Troels Kodal, Allen F. Shaughnessy, Dorte Qvesel

**Affiliations:** 1 Postgraduate Education, Medical Faculty, University of Southern Denmark, Denmark; 2 Department of Family Medicine, Tufts University School of Medicine, Boston, Massachusetts, U.S.A.; 3 Department of Postgraduate Medical Education, Southern Denmark, Denmark

**Keywords:** Postgraduate education, competency-based, foundation year, learning environment

## Abstract

Objectives: The purpose of this study is to explore the gains and losses, from the trainees’ perspective, that occur when replacing process-oriented basic medical training with competency-based training and to explore whether competency-based training can justify a reduction in clinical training.

**Methods:**

We performed a national cross-sectional survey of attitudes of Danish doctors who had completed either the old process-oriented 18-month training period (n=671) or the new competency-based 12-month training period (n=547). A total of 1218 doctors were included and 792 of them completed an online survey, yielding a response rate of 65%.

**Results:**

Trainees of the old process-oriented programme (53%) felt more ready to continue medical training than the doctors (84%) who followed the new and shorter competency-based programme. The differences was statistically significant (t _(790)_ = 11.16; p<0.0001). The latter group did not feel the competency-based programme improved the learning environment. Some trainees reported that learning objectives seem to optimize their learning within defined learning frames. They valued a curriculum that should not only contain learning objectives but that should also ensure relevant learning opportunities, providing sufficient time for learning and useful feedback.

**Conclusions:**

It is unlikely that a competency-based curriculum can justify a significant reduction in the time spent on clinical training. The learning approaches and the amount of time that we dedicate to training are important. Implementation of a new curriculum requires a substantial effort.

## Introduction

Outcome-based postgraduate education is an approach in which outcomes for learning are specified in terms of specific performance measures.^1^^-^^3^ It is a learner-centred approach that emphasizes attainment and documentation of performance in practice, called “outcomes.” It takes a constructivist approach and has the potential to decrease the time spent in training.^3^ In contrast, traditional, process-oriented training (PO) means education steered by defined learning frames and opportunistic learning rather than learning guided by objectives defining competency.^2^^,^^4^^,^^5^Until 2008, the postgraduate basic training programme in Denmark had a curriculum closely connected to 18 months’ mandatory training in homogeneous learning frames. The curriculum comprised 118 very specific objectives, but they were not given much focus in the clinical training^6^^,^^7^ and training was very process-oriented with assessments of learners based mostly on general impressions and not on the attainment of learning objectives.^8^A new curriculum was introduced in 2008. It focuses on 15 competencies important for the transition from medical school to clinical work. In contrast to the previous specific learning objectives, these competencies were general and specified performance-based measures, e.g., “The trainee should be able to perform follow-up consultations and adjust treatment in patients with chronic diseases.” One aim of this change in structure was to provide more supervision and feedback for trainees to strengthen the focus on outcome-based learning objectives.^9^ All trainees have to obtain the same general competencies, but each competency can be adapted to the specific learning situation found in the different departments. Local authorities chose the new enrolled departments.A second change was that the duration of the programme was reduced from 18 to 12 months. This reduction in training time was designed to be offset by the required formal assessment of each of the learning objectives to justify a reduction in training time without loss of qualifications. In 2009 both programmes were running simultaneously.The purpose of this study was to explore, from the trainees’ perspective, the gains and losses when replacing a process-oriented basic medical training programme with modern competency-based training, and more specifically to explore whether competency-based training can justify a reduction in clinical training.

## Methods

We conducted a national cross-sectional study of the attitudes and practices of trainees who completed either the 18-month process-oriented training period (PO) or the 12-month competency-based training period (CB) in 2009 and the first half of 2010 in Denmark. This study is part of a larger study and some results have been published elsewhere.^10^^,^^11^ A total of 1218 doctors were identified from the national database of all registered junior doctors, including 671 trainees in the 18-month PO programme and 547 trainees in the 12-month CB programme. The questionnaire was emailed to all participants nearing completion of their own postgraduate basic training. A reminder was sent three weeks later. The online questionnaire was developed to collect quantitative and qualitative data of trainees’ experiences and attitudes towards postgraduate basic training.^10^^,^^11^ The ten quantitative items were taken from the Postgraduate Hospital Educational Environment Measure (PHEEM) questionnaire^12^ and were rated on a 1-5 Likert scale (1=strongly disagree, 5=strongly agree). The questionnaire has been already validated in Danish.^12^ The items have also been used in general practice.^13^ In addition, an open-ended question was included in the questionnaire to examine trainees’ attitudes towards the ongoing change in postgraduate basic training.The internal construct reliability of the questionnaires was assessed with Cronbach's alpha analysis. The Alpha measure was 0.88.^14^ Confidence intervals of proportions were calculated and non-paired t-tests used to detect statistically significant differences (P values ≤0.05). The open-ended question was analyzed by empirical thematic analysis using a grounded theory approach.^15^ The responses were grouped into statements, which consisted of one or several sentences expressing one opinion. These statements were coded into categories and then condensed into themes. The identification of statements, coding, categorization and thematization were done by two researchers in collaboration. A third researcher finally approved the categorization and thematization independently. The categorization was only accepted if agreement among the three researchers was reached. A fourth researcher confirmed the conclusions.The two primary researchers had taken part in construction of the survey instrument, the third and the fourth researcher entered the process at the start of analysis. One researcher was part of the new CB curriculum team, one researcher was involved in the clinical implementation of the CB programme, one researcher has administrative responsibilities and the fourth researcher was an international researcher with no ties to Danish medical education. To minimize researchers’ bias, we enrolled researchers who had different professional backgrounds and different educational perspectives to provide triangulation.^16^ We also combined qualitative and quantitative data in the survey in order to apply the method of triangulation.^16^ We performed a pilot test in order to explore the level of comprehension of the questions. The first 200 responses were compared with the last 100 responses in each group in order to detect differences in early and late responders.Testing for data saturation was carried out by counting statements with new information from the second half responses and by ensuring the responders were representative of the entire year group of graduates. The internal validity of the survey instrument was improved by adjusting questions after the external review of the questionnaire. Six persons with comprehensive experiences of medical education and research provided constructive feedback to the questionnaire. These persons discussed identified categories to reach agreement on the final definition of each category or theme. External validity of the instrument was determined by comparing the qualitative results with the quantitative outcomes of the study and by comparing the results with other findings in the literature. The instrument has been published elsewhere.^11^

## Results

We received response from 792 of the 1218 trainees, 440 PO doctors and 352 GB doctors. An additional 38 trainees answered that they had not yet completed the training programme, e.g. due to maternity leave. Removing these trainees resulted in a response rate of 65%. Gender and geographic distribution of responders and non-responders were similar. (Table 1) There was no difference in the responses in the first 200 responses compared with the last 100 responses for both the groups.

**Table 1:** Demographics of respondents (N= 792)

Variable	N	%
Gender		
Male	278	35%
Female	514	65%
Response rate in different geographic regions		
Copenhagen	201	64%
Zealand	127	59%
South	199	69%
North	265	66%

### Quantitative analysis

In response to the item, ‘the training in this post makes me feel ready for continued specialisation’, PO trainees (53%) felt more ready to continue medical training than the CB trainees (84%) and the differences was statistically significant (t _(790)_ = 11.16; p<0.0001).

A further analysis of the CB responses from 2009 and 2010 showed no significant decrease from 54% in 2009 to 51% in 2010 (Table 2). Table 2 also shows the other results of the PHEEM survey. Trainees were asked to rate their overall view of “the ability to utilize learning opportunities” in internal medicine wards. There was no other significant difference in junior doctors’ perception during the PO and the CB programme in the other closed ended items.

**Table 2:** Comparison of mean and standard deviation (SD) of the old (PO) and new (CB) programme (N= 792)

Items*	I had an informative Introduction programme Mean(SD)		My clinical teachers provided me with good quality feedback on my strengths and weaknesses Mean(SD)		Senior staff utilized learning opportunities effectively Mean(SD)	
Programme	PO	CB	PO	CB	PO	CB
Internal medicine	3.6 (1.19)	3.5 (1.13)	3.2 (1.13)	3.2 (1.12)	3.3 (1.10)**	3.0 (1.09)**
Surgery/ orthopaedics	3.2 (1.12)	3.2 (1.12)	2.9 (1.03)	2.9 (1.11)	3.0 (1.11)	3.0 (1.12)
General practice	4.0 (0.94)	4.0 (1.01)	3.8 (1.00)	3.9 (1.00)	4.0 (0.98)	3.9 (1.01)

*Trainees rated their agreement on a 5-point Likert scale from 1 (strongly disagree) to 5 (strongly agree).

**t (585) = 1.64; p=0.05

### Qualitative analysis

We received 702 qualitative responses, 378 from PO trainees and 324 from CB trainees. The responses from the trainees contained from one to eight statements. The 3011 statements were coded into six categorises. Seventy-nine statements were unintelligible or ambiguous and therefore not coded (Table 3).

**Table 3:** The categorisation of responses

1	Attitudes about curriculum, medical competencies and quality in education
2	Problems / advantages of the participating wards, and time used for training
3	Non-specified positive or negative remarks
4	Identity as a doctor
5	Collaboration in healthcare
6	Social geographic issues and economy

After coding approximately half of the statements, no new information came up in the remaining statements. General statements concerning social, economic and geographic issues and non-specific positive or negative remarks were excluded. The remaining four categories with 2659 statements were condensed into three themes: 1) importance of learning frames and time spent in clinical training; 2) attitudes towards learning objectives; and, 3) need for self-defined learning objectives. There was remarkable unanimity between the responders from the PO and the CB programme.

### Importance of learning frames and time spent in clinical training

According to the responding trainees, it is the type and number of clinical tasks performed and the degree and quality of supervision, which determines the increase in competence. Trainees trained within different clinical settings will achieve different outcomes even though they are following the same curriculum.

The trainees expected that an intensified focus on learning objectives could facilitate learning but that it would not compensate for the 6-month reduction in time used for clinical training. The CB doctors stated that several of the wards now taking part in the postgraduate basic training programme did not provide learning frames suitable for their training.

The doctors stated that a learning frame is much more than just a “type of ward”, a learning frame depends on the type of clinical tasks and functions that the junior doctors were allowed to train on in the specific ward.

*“I think it was very beneficial to be trained in three major specialties (internal medicine, surgery and general practice) I felt competent and ready to start in any specialist training programme.”* PO trainee

*“...The reduction in time makes me feel like a slightly amputated doctor...I miss a more all-round introduction to clinical practice.”* CB trainee

*“...By leaving out departments such as internal medicine you reduced the needed outcome considerably despite the new curriculum.”* CB trainee

### Attitudes towards learning objectives

Both the CB and the PO trainees were positive about the strengthened focus on learning objectives and especially about the intended associated feedback and supervision. The trainees following the new CB programme stated, however, that the wards continued “business as usual” in the clinical training with little or no attention given to the focus on new learning objectives and feedback.

The CB trainees were not pleased with the general competencies defined in the new curriculum. They requested more specific objectives aimed at the clinical tasks they had to perform in their present position. General competencies provided them with fewer guarantees for proper training than more specific learning objectives associated with specific learning frames.

“The learning goals are too vague and imprecise. They need to be much more concrete and specific. I think it is very inappropriate that we are not trained in the same type of ward and that we are not all trained at the internal medicine ward.” CB trainee

### Need for self-defined learning objectives (hidden curriculum)

The trainees stated that they defined their own extra objectives. These objectives could be categorized as specific medical competencies, intermediate competencies, gaining horizontal expertise, and ability to collaborate. The specific medical competencies were aimed at dealing with the medical conditions they expected to encounter in the near future. The intermediate objectives were found necessary in order to gain access to interesting clinical work on the wards. Another type of objective was “broad basic medical knowledge” or “general problem-solving skills,” knowledge and skills they found necessary, when dealing with unclear medical complaints. The ‘ability to collaborate’ objectives involved working in a team and working in health care organization, as well as understanding the working conditions of colleagues, both at major hospitals wards and in general practice.

*“You need knowledge about how to treat common medical conditions such as lung oedema, asthma and pneumonia.”* CB trainee

*“You need a broad understanding of medical diseases in order to deal with unclear clinical manifestations and avoid unnecessary referrals.”* CB trainee

*“…a stay in general practice is necessary for all doctors because you need to know how the (healthcare) system works.”* CB trainee

It appears that the PO trainees had more confidence in their own skills in comparison with the CB trainees.

## Discussion

The transition from a traditions postgraduate training programme to competency based training open a unique opportunity to explore gains and losses, from the trainees’ point of view, in comparison to competency based training.

In this study we found that the introduction of a 33% shorter competency-based postgraduate training programme resulted in trainees who felt less qualified. Further, we found that the introduction of a competency based curriculum had no positive impact on the learning environment as perceived by the trainees.

The junior doctors felt that learning outcomes were very context-specific. They welcomed a new curriculum because they hoped it would strengthen supervision and feedback. They were, however, very concerned about the reduction in time and the lack of flexibility in working on participating wards. According to the young doctors a curriculum should do more than provide relevant objectives, it should also ensure good learning frames, proper learning opportunities, sufficient time for training and feedback. The trainees appreciated help in focusing on the relevant learning opportunities within proper clinical settings and feedback. The trainees preferred specific objectives to more general competencies in the curriculum. The study highlighted that the introduction and implementation of a new competency-based curriculum is a challenge and requires significant effort.

### Strengths and limitations of this study

The strength of this study is that we used a previously validated survey along with a qualitative element to obtain trainees’ opinions regarding their medical preparation. We had a 65% response rate that, while not ideal, is representative of the complete population given the gender and geographic distribution of the respondents.

Ideally, we would have been able to randomly assign training sites and trainees before conducting this project. However, we have no reason to believe there was a systematic bias in resident selection of training site that would have affected our results. The use of an online questionnaire, which collects quantitative and qualitative data, gives rise to methodological considerations. The quantitative items have earlier been used in Danish and found applicable.^12^^,^^13^ The quantitative responses in the last 100 responses showed no significant difference to first 200 responses in each group, indicating that early and prompt responders and late responders share the same attitudes.

The reported level of doctors’ competencies is based on self-evaluation and is not on an objective measurement and the correlation between self-evaluation and objective measurement is questionable on an individual level.^18^ Since the number of responders is high, however, we assume that the average of the self-evaluations reflect the level of obtained competence in the two groups.

Applying qualitative analysis on data obtained from open-ended items from a questionnaire has been used earlier^23^ but it is not well supported in the qualitative methodology literature.^17^ Caution should therefore be taken with the interpretation of the data. However, we received a high number of long and reflective statements from a very broad, representative group of Danish trainee doctors and we applied a systematic approach to data analysis. We therefore think the statements provide us with comprehensive information about the attitudes of trainee doctors.

We asked a general open-ended item about doctors’ attitudes towards the ongoing change in the postgraduate basic training. Specific questions could have provided a more comprehensive description of the attitudes of doctors concerning specific topics. We analysed the spontaneous statements generated after reflecting on the advantages and disadvantages of the educational restructuring. We therefore expect that the statements refer to issues the junior doctors find most important. No new information came up in the second half of the qualitative responses. We therefore assume we have obtained data saturation. By combining quantitative and qualitative data it is possible to combine the ratings with exploratory statements, which could be considered to be a strength in this study. We have in this study only focused on the perceptions of junior doctors who had just finished their basic training. Data from senior doctors and from junior doctors later in their specialist training may challenge the importance of the expressed ratings and attitudes.

Uncertainty in connection with programme change may bias the statements. New initiatives need time to be properly implemented. On the other hand, no change was observed in the statements and ratings between the CB doctors from 2009 and 2010.

There was consistency in responses from doctors training before, during and after the educational change. We therefore expect that our findings have high generalisability regarding Danish junior doctors and trainees with similar cultural and educational background and with similar training conditions.

### Comparison with existing literature

In the literature, hope has been expressed that a comprehensive focus on the requested competencies would allow more flexible views on time needed for training, number of learning opportunities and type of learning environment.^3^^,^^20^ The trainees in this study were however reluctant towards the reduction in time and the de-contextualization of training. The necessity of specific and sufficient learning opportunities can be supported by the literature.^4^^,^^10^^,^^20^^-^^22^The trainees preferred specific objectives to more general competencies in the curriculum, which is in contrast to the approach advocated for outcomes-based education.^2^^,^^3^ Their views are however contradicted by their self-defined learning objectives, which were relatively general. One explanation could be that the doctors in this study are young and newly graduated and they may therefore have difficulty grasping the full concept of a general competence. They may not value the attainment of cognitive skills embodied in the general competency as much as they do the accumulation of specific knowledge.Learners may feel, though, that a busy ward needs specific learning objectives, with specific training strategies and feedback to ensure proper training for them. General competency statements may not provide sufficient structure to ensure specific training. Young doctors see the curriculum more as a training warranty than as an assessment and learning tool. They believe their learning may be impaired if the needed learning frames are not properly defined via learning objectives. This focus on specific objectives could be very developmentally appropriate, when early learners want to know rules gained through experience about a wide variety of illnesses.It is, however, a challenge to ensure that a focus on very specific objectives does not disturb the development of clinical competence.^3^^,^^21^^,^^26^ Ideally, a curriculum with integrated comprehensive educational plans describing optimized learning frames, sufficient leaning opportunities and reflective supervision would ease the need for a training guarantee and allow the trainees to get a more positive view on general competencies containing metacognitive elements.^3^^,^^27^ It has earlier been shown that easy access to a curriculum helps doctors get a better focus on relevant learning objectives in a sea of incalculable learning possibilities.^23^ Trainees seem to redefine and extend the official learning objectives. However this should not be seen as negative, rather it shows the ability to reflect and perform self-directed learning.^24^^, ^^25^

### Implications

When moving away from a one-sided, process-oriented programme, care should be taken to avoid ending up with a one-sided, competency-based programme with too much focus on requested learning outcomes and too little focus on the needed learning opportunities.Comprehensive implementation of a formal curriculum into busy clinical wards is a challenge.^28^ Our data indicate that a healthcare system should not assume that a new competency-based curriculum can justify a reduction in time spent on clinical training without loss of perceived competence – unless it contains a very comprehensive implementation strategy based on a very accurate and rigorous analysis of needed learning frames, learning opportunities and time.The hopes from the Danish health authorities that competency-based basic training, could justify a 33% reduction in time spent on training and that general competence could be attained in very different clinical settings cannot be supported by this study.More research on how curricula affect junior doctors’ outcomes is needed, e.g. from a senior doctor’s perspective, and on how to successfully implement curriculum changes in busy clinical workplaces.

### Conflict of Interest

The authors declare that they have no conflict of interest.
